# Nasal Polyp-Derived Mesenchymal Stromal Cells Exhibit Lack of Immune-Associated Molecules and High Levels of Stem/Progenitor Cells Markers

**DOI:** 10.3389/fimmu.2017.00039

**Published:** 2017-01-30

**Authors:** Pedro Wey Barbosa de Oliveira, Rogério Pezato, Juan Sebastian Henao Agudelo, Claudina Angela Perez-Novo, Wim Vanden Berghe, Niels Olsen Câmara, Danilo Candido de Almeida, Luís Carlos Gregorio

**Affiliations:** ^1^ENT Research Laboratory, Department of Otolaryngology-Head and Neck Surgery, Federal University of São Paulo, São Paulo, Brazil; ^2^Department Biomedical Sciences, University of Antwerp, PPES Lab Proteinchemistry, Proteomics Epigenetic Signaling, Wilrijk, Belgium; ^3^Department of Medicine, Nephrology Division, Federal University of São Paulo, São Paulo, Brazil; ^4^Department of Immunology, Institute of Biomedical Sciences, University of São Paulo, São Paulo, Brazil

**Keywords:** mesenchymal stromal cells, nasal polyposis, gene expression, immunoregulation, stem cells

## Abstract

Mesenchymal stromal cells (MSCs) are considered adult progenitor stem cells and have been studied in a multitude of tissues. In this context, the microenvironment of nasal polyp tissue has several inflammatory cells, but their stroma compartment remains little elucidated. Hence, we isolated MSCs from nasal polyps Polyp-MSCs (PO-MSCs) and compared their molecular features and gene expression pattern with bone marrow-derived MSCs (BM-MSCs). Initially, both PO-MSCs and BM-MSCs were isolated, cultivated, and submitted to morphologic, differentiation, phenotypic, immunosuppressive, and gene expression assays. Compared to BM-MSCs, PO-MSCs showed normal morphology and similar osteogenic/adipogenic differentiation potential, but their immunophenotyping showed lack of immune-associated molecules (e.g., CD117, HLA-DR, PDL-1, and PDL-2), which was linked with less immunoregulatory abilities such as (i) inhibition of lymphocytes proliferation and (ii) regulatory T cell expansion. Furthermore, we detected in the PO-MSCs a distinct gene expression profile in comparison with BM-MSCs. PO-MSC expressed higher levels of progenitor stem cells specific markers (e.g., CD133 and ABCB1), while BM-MSCs showed elevated expression of cytokines and growth factors (e.g., FGF10, KDR, and GDF6). The gene ontology analysis showed that the differentially modulated genes in PO-MSC were related with matrix remodeling process and hexose and glucose transport. For BM-MSCs, the highly expressed genes were associated with behavior, angiogenesis, blood vessel morphogenesis, cell–cell signaling, and regulation of response to external stimulus. Thus, these results suggest that PO-MSCs, while sharing similar aspects with BM-MSCs, express a different profile of molecules, which presumably can be implicated in the development of nasal polyp tissue.

## Introduction

Mesenchymal stromal cells (MSCs) are considered adult progenitor stem cells and have been studied in a set of pro-regenerative studies (1). MSCs are multipotent cells, with ability to differentiate into mesodermal cell lines (e.g., chondrocytes, adipocytes, and osteocytes) and can be obtained from several tissues (e.g., bone marrow, adipose tissue, umbilical cord, and muscle) (2); however, their presence in tissues affected by intense inflammation, as polyps, remains poorly elucidated.

Nasal polyposis (NP) is a chronic inflammatory condition of the upper airways, characterized by an overgrowth of paranasal sinus mucosa, with increase in eosinophil infiltration and high levels of interleukin 5 and eosinophil cationic protein (3, 4). The inflammatory reaction involves several cell types in NP and is primarily driven by a T helper-2 response (5, 6). Two major factors are related to nasal polyp formation: an abnormal remodeling response, creating a mechanical dysfunction (7, 8) and a lack of immune regulatory effects, favoring a severe inflammatory process (9).

In this sense, MSCs have a great therapeutic potential, showing specific immunomodulatory effects and an ability to directly or indirectly modulate the fibrotic process (10–12). These two MSCs abilities could have an immediate impact on NP, mitigating the tissue inflammation and rebalancing the remodeling process. In this context, our group and others’ recent studies have demonstrated a potential role of bone marrow-derived MSCs (BM-MSCs) in the modulation of several immune cells in inflamed nasal polyp tissue (13, 14). Considering this perspective, MSCs derived from nasal polyps could show different features, which would participate in the regulation of NP microenvironment, thus eliciting favorable conditions for polyp development.

Hence, in this study, we isolated MSCs from nasal polyp tissue [Polyp-MSCs (PO-MSCs)] and pointed out their main characteristics in comparison to classically known BM-MSCs. We demonstrated that PO-MSCs share similar aspects with classical BM-MSCs but have a different gene expression profile, which is associated with signaling pathways linked to stem cell biology, metabolic processes, and matrix remodeling.

## Materials and Methods

### MSC Isolation and Culture

The mononuclear cells for BM-MSCs isolation were collected from healthy donors at the Support Group for Children and Adolescents with Cancer in the Children’s São Paulo Hospital (GRAAC), after ethical approval and donors’ consent (*n* = 6, protocol no. 45/09, accession number: *30540214.0.0000.5505*). PO-MSCs were collected after endoscopic polypectomy surgery in patients with NP (*n* = 4), according to ethical approval and donors’ consent at São Paulo Hospital, number: *EPOS 12* (3). Both MSC subtypes were isolated according to Pezato et al. (13). Briefly, the transplantation plastic filters containing bone marrow cells were washed in PBS solution, and the cells were isolated using Ficoll-Hypaque method (Sigma, USA). The PO-MSCs were isolated from nasal polyp tissues by mechanical dissociation (using forceps and scissors), followed by 50 min of enzymatic digestion at 37°C (collagenase IV 1 mg/mL, Sigma). Both cells were washed in sterile PBS and filtered in a 70-μm filter (BD Biosciences, USA). After, both MSC subtypes were suspended and cultivated in 25 cm^2^ culture flasks (Corning, NY, USA) at 37°C in D-MEM low-glucose culture medium (45 mM NaHCO_3_, 10% FBS, 100 U/mL penicillin, 100 U/mL streptomycin, Gibco, USA) in a humidified atmosphere and 5% CO_2_.

### *In Vitro* Differentiation Assays

The *in vitro* multipotent differentiation potential into mesenchymal lineages (i.e., adipocytes and osteoblasts) was assessed using the adipogenesis and osteogenesis Mesenchymal Stem Cell Kit (Millipore, USA), according to the manufacturer’s specifications.

### Immunophenotyping

The immunophenotyping of both different types of MSCs was carried out using a specific set of antibodies (i.e., CD34, CD45, CD105, CD90, CD73, CD54, CD117, HLA-DR, PDL-1, and PDL-2, BD Bioscience, USA), according to the manufacturer’s recommendations. The cells were collected, and the immunostaining was adjusted to 1:100 of antibody dilution. Then, the cells were incubated with a specific antibody per 30 min at room temperature in FACs buffer (PBS + 2% FBS). Then, the cells were washed in PBS solution and suspended in FACs buffer for acquisition in a flow cytometer. The FACs Canto II (BD, Beckton Dickson) was used for cell acquisition, and the FlowJo software was used for data collection and analysis.

### Lymphocyte Proliferative Assay and Treg Expansion

For investigate the immunosuppressive potential of PO-MSCs and BM-MSCs, these cells were cultivated with fresh peripheral blood mononuclear cell (PBMC) in two different lymphocytes/MSCs proportions: (i) 5:1 and (ii) 20:1. The lymphocytes were isolated from healthy donors by Ficoll-Hypaque method (Sigma, USA) and previously labeled with fluorescent dye, Cell Trace (Life Technologies, USA), following the manufacturer’s instructions. For proliferation assay, PBMCs were cultivated by 6 days with or without MSCs (from Polyp or BM) under anti-CD3/CD28 stimulus (1/2 μg/mL, respectively) with RPMI medium + 10% FBS in flat bottom 96-well plate (TPP, USA). Then, all non-adherent cells were collected, stained with anti-Foxp3, anti-CD4, and anti-CD8 conjugated antibodies (APC, FITC, and Percep, BD, Beckton Dickson) and subsequently analyzed by flow cytometry following protocols and acquisition parameters aforementioned.

### Gene Expression Profile of MSCs and *In Silico* Analysis

Total RNA from both bone marrow and nasal polyp MSC cells was extracted using an RNeasy Mini Kit (50) (Qiagen, South Korea), according to the manufacturer’s instructions. The concentration and integrity of RNA samples were, respectively, evaluated using a Nanodrop spectrophotometer (Thermo Scientific, USA) and 1% agarose gel electrophoresis (Gibco, USA). Furthermore, reverse transcription of total RNAs was performed using the RT^2^ First Strand Kit (Qiagen, South Korea). Global gene expression profile was performed in 96-well plates per each set, following the recommendations specified in the products’ catalogs: RT^2^ SYBR Green ROX qPCR Master Mix and Mesenchymal Stem Cells PCR array (84 genes; Qiagen, South Korea). Data analysis and normalization were performed using the free online software provided on the manufacturer’s website (Qiagen, South Korea). The signaling pathways associated with differentially modulated genes were performed in the *GOminer* web software (website or reference), according to the specificity in the default layout. The transcriptional factor enrichment analysis was carried out using the functional enrichment analysis web tool *FUNRICH* using standard default.

### Statistical Analysis

All statistical analysis and graphs were performed using the GraphPad Prism 5 software (GraphPad, Inc., USA). Data were presented according to classical descriptive statistics. Results were tested for normal distribution by the Kolmogorov–Smirnov test with Dallal–Wilkinson–Lillie for a *p*-value. Categorical variables were expressed as percentages (%), and continuous variables (data) were presented as means ± SDs. The parametric Student’s *t*-test and One-way ANOVA test were used to assess differences between groups. For all analyses, a *p*-value ≤0.05 was considered statistically significant.

## Results

### *In Vitro* Characterization and Immunophenotyping of PO-MSCs and BM-MSCs

**Figure 1:**
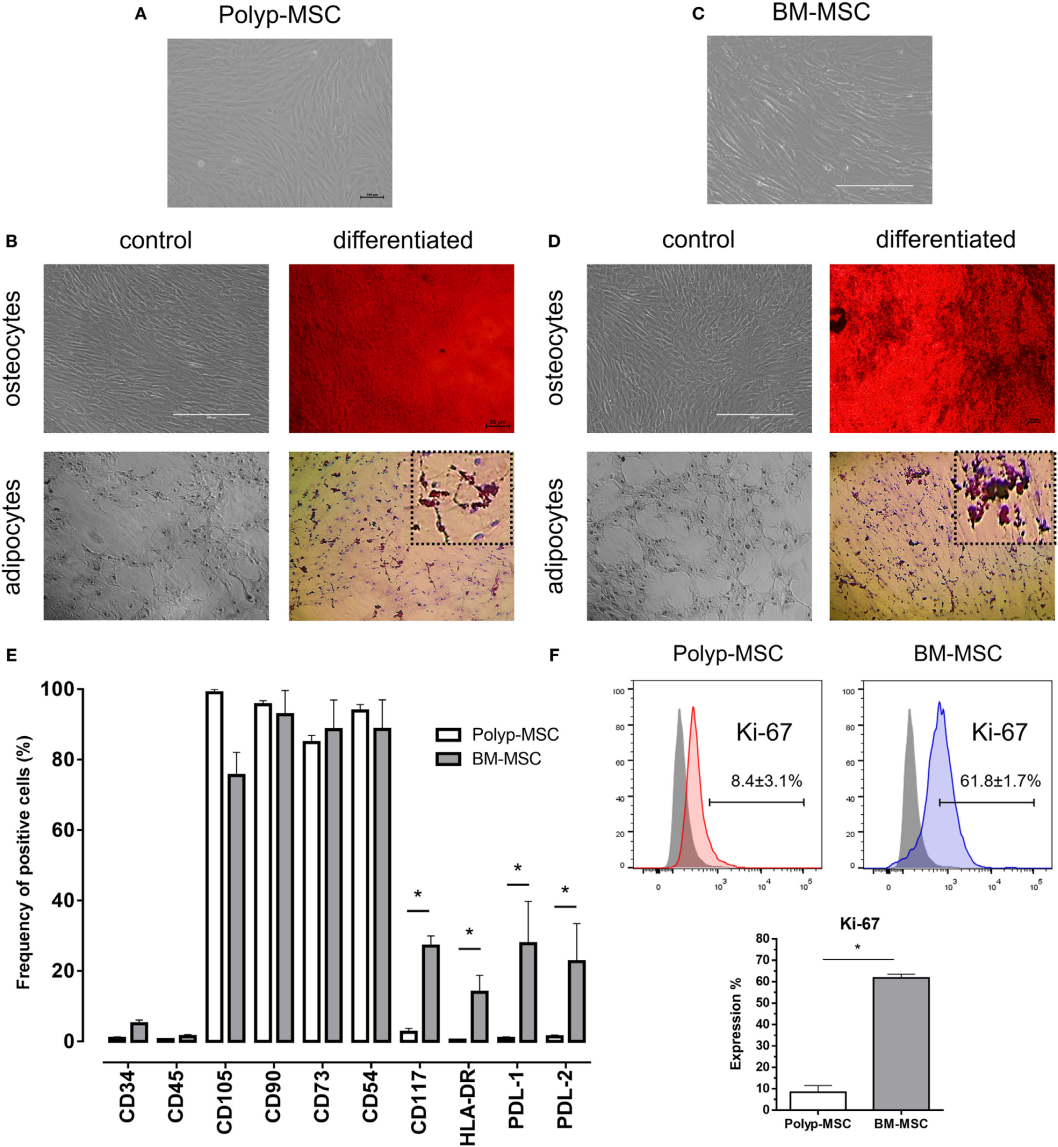
**Characterization of Polyp-MSCs (PO-MSCs) and bone marrow-derived mesenchymal stem cells (BM-MSCs)**. **(A)** Fibroblast-like morphology of PO-MSCs; **(B)** mesodermal differentiation potential of PO-MSC; **(C)** fibroblast-like morphology of BM-MSCs; **(D)** mesodermal differentiation potential of BM-MSC; **(E)** immunophenotyping of mesenchymal stromal cells derived from polyp and bone marrow tissues; and **(F)** Ki-67 expression in PO-MSC and BM-MSCs. A similar morphology and differentiation potential into adipocyte- and osteocyte-like cells were observed in cultures of PO-MSCs and BM-MSCs. However, the PO-MSCs in comparison with BM-MSCs showed a decreasing in the expression of surface molecules related to immunoregulation (i.e., CD117, HLA-DR, PDL-1, and PDL-2) and proliferation (Ki-67) (*p < 0.05).

First, both MSC subtypes were isolated and submitted to standard culture conditions (see Figure S1 in Supplementary Material). These cells had fibroblastic-like morphology (Figures 1A,C) and similar differentiation potential into mesodermal lineages such as adipocytes and osteocytes (Figures 1B,D). The immunophenotyping analysis of PO-MSCs and BM-MSCs showed a negative expression for hematopoietic surface markers (CD34 and CD45) and a positive status for classical mesenchymal markers, such as CD105, CD90, CD73, and CD54 (Figure 1E; Figure S2 and Table S1 in Supplementary Material). However, the PO-MSCs in comparison with BM-MSCs had reduced expression of molecules related to immune regulation process, e.g., CD117, HLA-DR, PD-L1, and PD-L2 (Figure 1E; Figure S2 and Table S1 in Supplementary Material). The internal proliferation rates did not change along the passages showing no significant difference (data not showed); however, the global intranuclear Ki-67 expression (a proliferation pan marker) of BM-MSCs was higher than PO-MSCs (Figure 1F), suggesting that BM cells can present more proliferative status.

### Immunosuppressive Assay of PO-MSCs and BM-MSCs in Coculture with CD4^+^ and CD8^+^ Lymphocytes

**Figure 2:**
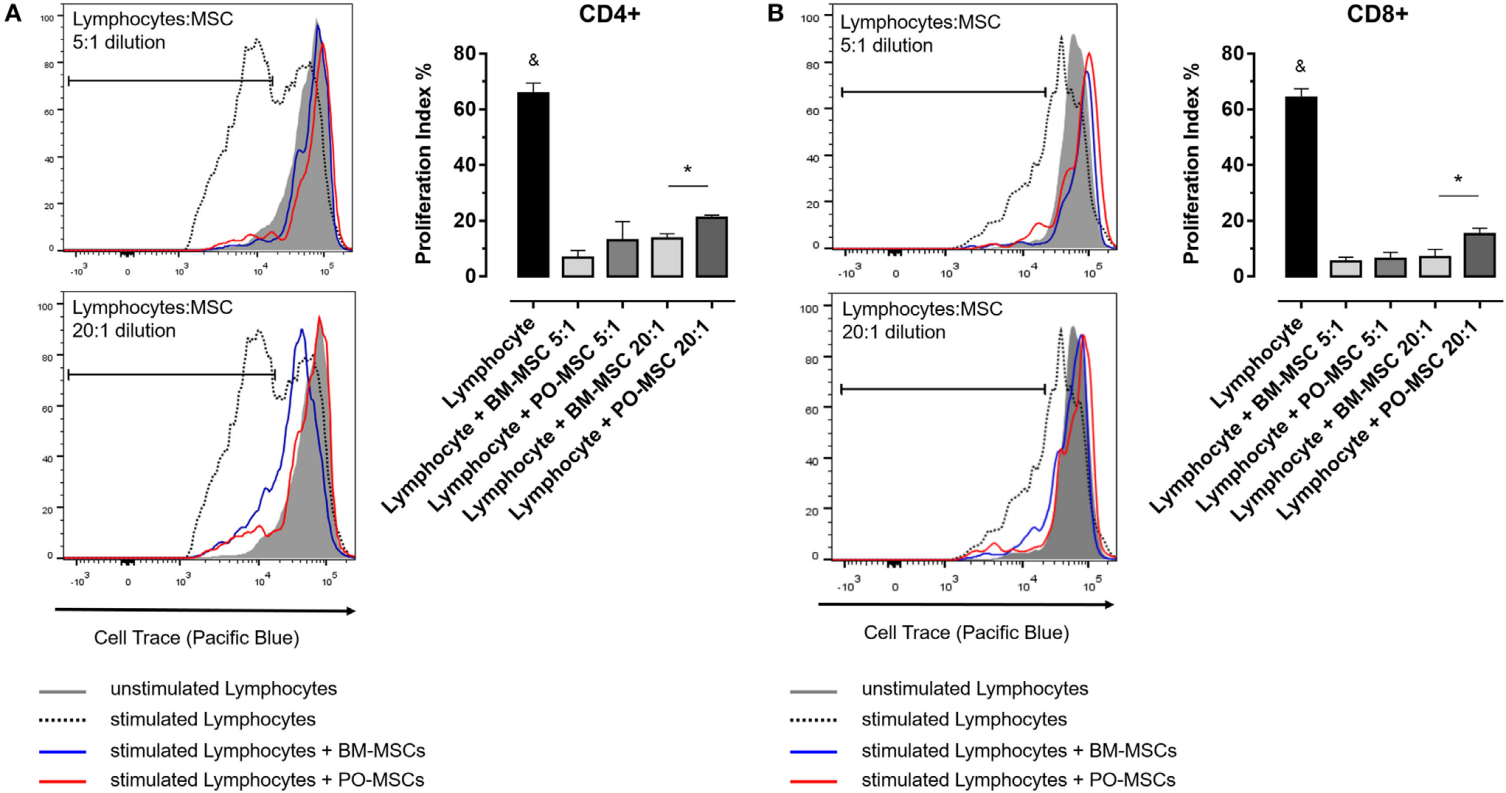
**Lymphocyte proliferation assay in coculture with Polyp-MSCs (PO-MSCs) and bone marrow-derived MSCs (BM-MSCs)**. **(A)** Lymphocytes were stimulated with anti-CD3/CD28 antibodies per 6 day and were cocultivated in presence of PO-MSCs or BM-MSCs in two different dilutions with 5:1 and 20:1 of lymphocytes/MSCs proportion. **(A,B)** Refer, respectively, to proliferation index of CD4^+^ and CD8^+^ lymphocytes in different conditions [i.e., without mesenchymal stromal cells (MSCs), with MSCs at 5:1 and 20:1 dilutions]. It was observed that both MSCs inhibited the total lymphocytes proliferation; however, PO-MSCs in comparison with BM-MSC had less immunosuppressive response at higher dilution (e.g., 20:1 proportion) (^&^*p* < 0.05 in comparison to all groups and *p < 0.05 in comparison to BM-MSC in 20:1 dilution with lymphocytes).

**Figure 3:**
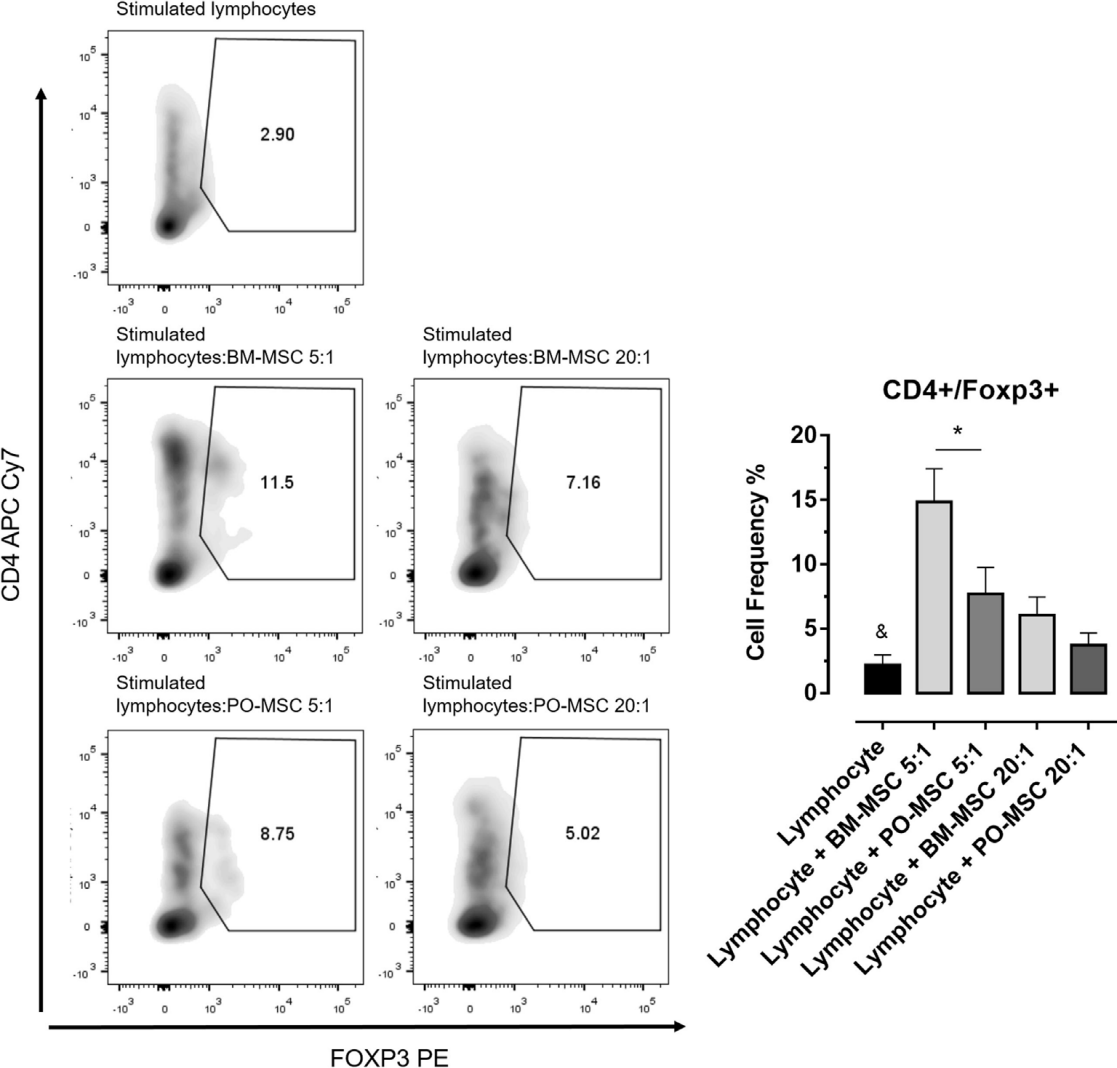
**Frequency of regulatory T cell (Treg) (CD4^+^/Foxp3^+^) in coculture with Polyp-MSCs (PO-MSCs) and bone marrow-derived MSCs (BM-MSCs)**. During coculture assay with CD4^+^ lymphocytes and mesenchymal stromal cells (Polyp and BM), it was possible to detected that only BM-MSC had strong abilities to promote the expansion of Treg population (CD4^+^/Foxp3^+^) at 5:1 dilution. Although PO-MSCs have expanded Tregs, its index was inferior to BM-MSC considering the 5:1 and 20:1 dilutions (^&^*p* < 0.05 in comparison to all groups and *p < 0.05 in comparison to BM-MSC in 5:1 dilution with lymphocytes).

In order to elucidate the immunoregulatory potential of PO-MSCs, we performed a coculture assay of these cells with T lymphocytes and compared its functionality with BM-MSCs, which classically present strong immunomodulatory abilities. First, we observed that lymphocyte alone showed higher proliferation index (>60%) (Figure 2). After, we detected at two different dilutions (i.e., 5:1 and 20:1 lymphocytes/MSCs proportion) that both MSC subpopulations (PO-MSCs and BM-MSCs) presented capacities to suppress CD4^+^ and CD8^+^ lymphocytes proliferation; however, the immunosuppressive potential was inferior to PO-MSC when compared with BM-MSC, considering the 20:1 proportion (Figure 2). Interestingly, when we observed in this same coculture the frequency of CD4^+^/Foxp3^+^ cells, a subpopulation of regulatory T cell (Treg), its index was higher in BM-MSC presence than PO-MSC, considering the 5:1 proportion (Figure 3). However, no difference in Tregs frequency was verified in higher dilution (20:1 proportion) at both MSC populations: PO-MSCs and BM-MSCs (Figure 3).

### Analysis of the Gene Expression Profile of PO-MSCs in Comparison with BM-MSCs

**Figure 4:**
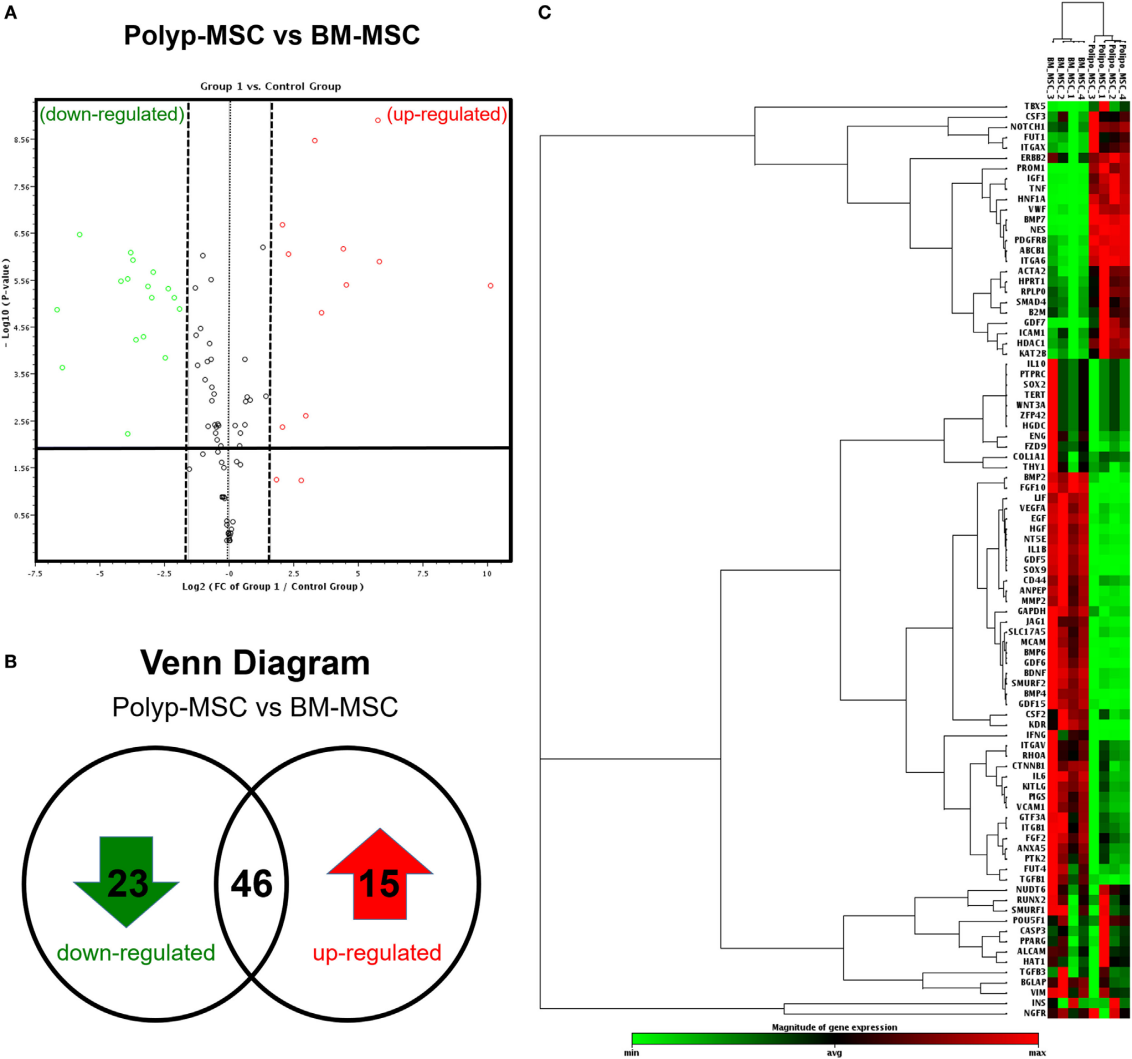
**Global gene expression profile**. **(A)** Volcano-plot graph showing the most representative upregulated (red) and downregulated (green) genes in Polyp-MSC (PO-MSC) in comparison with bone marrow-derived MSC (BM-MSC); **(B)** Venn diagram illustrating the number of upregulated/downregulated genes; and **(C)** heat map-cluster analysis demonstrating a global difference in gene expression, as well as the most representative clusters of samples and genes. Interestingly, there were several modulated genes in the comparison of PO-MSCs versus BM-MSCs, and the mesenchymal stromal cells from each tissue (Polyp and bone marrow) showed a different profile, being grouped in the same group.

To improve our extensive characterization between these two MSC subpopulations derived from different tissues, we performed the global gene expression profile, covering 84 genes associated with mesenchymal stem cell biology. The volcano plot analysis showed the significant modulation of specific genes, which were up- and downmodulated in the PO-MSCs in comparison with BM-MSCs (Figure 4A). More specifically, using the *Venn Diagram* strategy, we detected 15 upregulated genes, 23 downregulated genes, and 46 genes similarly expressed in PO-MSCs when compared with BM-MSCs (Figure 4B; Figure S3A in Supplementary Material). Subsequently, using heat map analysis, it was possible to observe the global differences in gene expression profile (green/down and red/up colors) between the two assessed cell types, as well as the most representative clustering groups of samples and genes for each cell preparations (Figure 4C; Figure S3B in Supplementary Material). Interestingly, several genes were modulated in the comparison of PO-MSCs versus BM-MSCs, and the samples of each different tissue were grouped in distinctive clusters, demonstrating a similar transcriptional pattern between samples derived from polyp tissues and bone marrow tissues (Figure 4C).

### PO-MSCs and BM-MSCs Have Different Transcriptional Profiles

**Figure 5:**
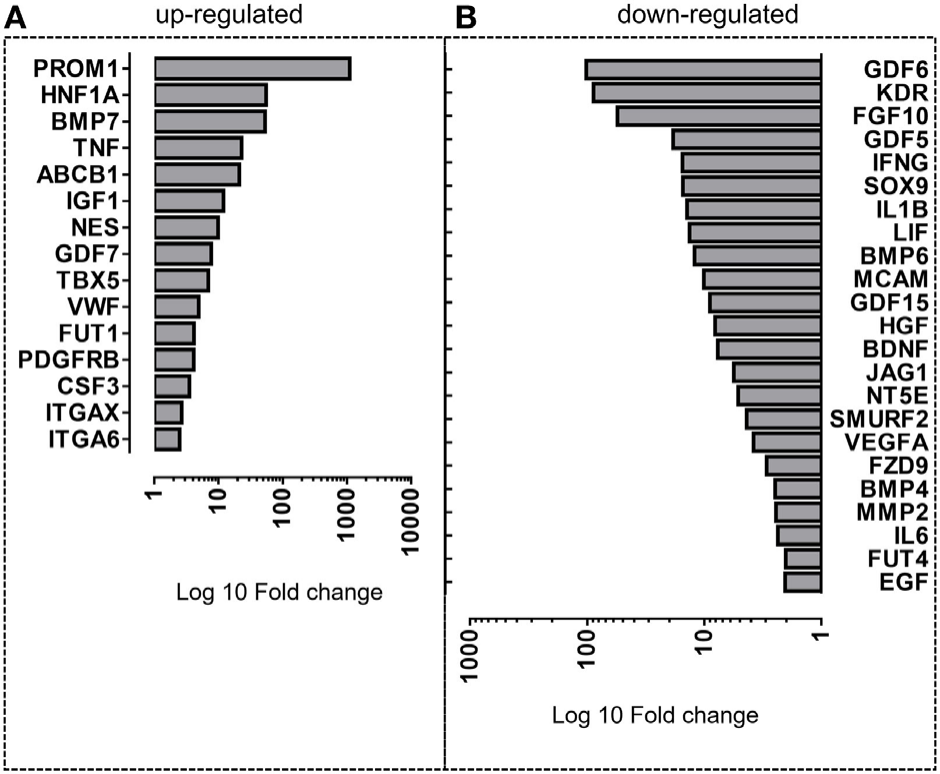
**Differentially expressed genes in Polyp-MSCs (PO-MSCs) in comparison with bone marrow-derived MSCs (BM-MSCs)**. Bar plot representing the most upmodulated **(A)** and downmodulated **(B)** genes in the comparison of PO-MSC versus BM-MSC. A differential gene expression profile is observed between PO-MSCs and BM-MSCs, suggesting a distinct transcriptional background for these cell populations.

In an attempt to elucidate the differentially modulated genes in the PO-MSCs in comparison with BM-MSCs, we carried out the fold-change analysis and pointed out the most significant upregulated or downregulated genes. For PO-MSCs, we found *PROM1, HNF1A, BMP7, TNF, ABCB1, IGF1, NES, GDF7, TBX5, VWF, FUT1, PDGFRB, CSF3, ITGAX*, and *ITGA6* as the most often expressed genes, and for BM-MSCs, we detected *GDF6, KDR, FGF10, GDF5, IFNG, SOX9, IL1B, LIF, BMP6, MCAM, GDF15, HGF, BDNF, JAG1, NT5E, SMURF2, VEGFA, FZD9, BMP4, MMP2, IL6, EGF*, and *FUT4* as the most upregulated ones (Figure 5). Surprisingly, the PO-MSCs in comparison with BM-MSCs showed higher expression of PROM1 or CD133 and ABCB1, which are considered progenitor markers, suggesting a different stem cell property for this MSC subtype. On the other hand, the BM-MSCs showed a strong regulation of genes associated with cytokines and growth factors (i.e., FGF10, KDR, and GDF6), which are particular for these BM-MSC subsets (Figure 5).

### Signaling Pathways Associated with Upregulated Genes in PO-MSCs and in BM-MSCs

**Figure 6:**
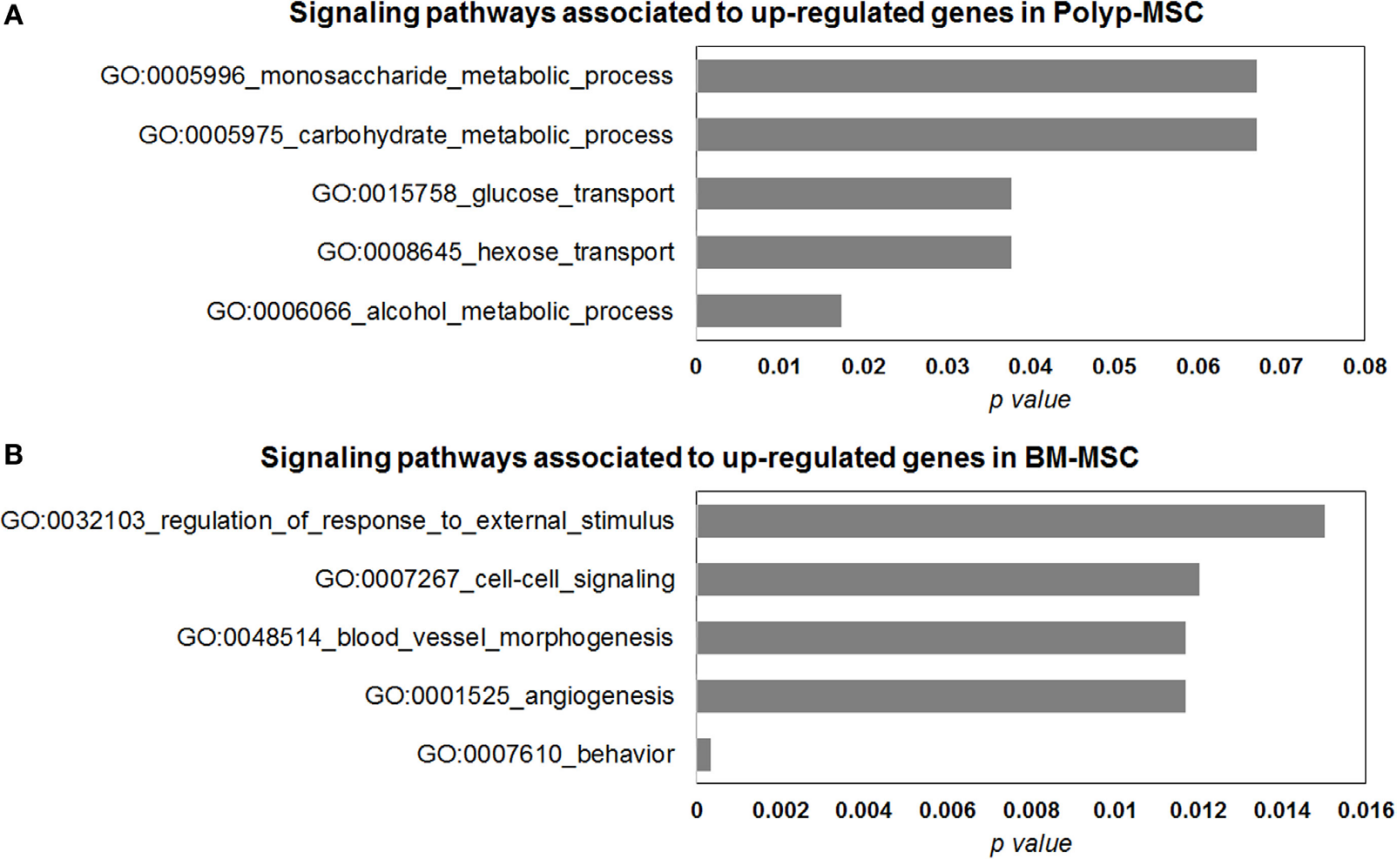
**Gene ontology analysis of differentially modulated genes in Polyp-MSCs (PO-MSCs) in comparison with bone marrow-derived MSCs (BM-MSCs)**. **(A,B)** Gene ontology analysis showing genes regulated (up/down) in PO-MSCs in comparison with BM-MSCs, which are predicted to be enriched in different signaling pathways. For the genes upregulated in PO-MSC, an association was found with alcohol metabolic process, hexose transport, glucose transport, carbohydrate metabolic process, and monosaccharide metabolic process, while for genes upregulated in BM-MSCs, the association was linked to behavior response, angiogenesis, blood vessel morphogenesis, cell–cell signaling, and regulation of external stimulus response.

To elucidate some interesting signaling pathways associated with most upmodulated/downmodulated genes in PO-MSCs in comparison with BM-MSCs, we performed the gene ontology analysis and searched for biological pathways that were significantly enriched with those genes. Using this *in silico* analysis, we detected alcohol metabolic process, hexose transport, glucose transport, carbohydrate metabolic process, and monosaccharide metabolic process as most significant biological pathways associated with upregulated genes in PO-MSCs (Figure 6A). In contrast, for BM-MSCs, we found behavior, angiogenesis, blood vessel morphogenesis, cell–cell signaling, and regulation of external stimulus response as the most enriched signaling pathways linked to upregulated genes. Altogether, these findings suggest that PO-MSC and BM-MSC are different MSC cell subpopulations, showing distinct transcriptional background, which can reflect their specific biological properties for each tissue’s localization (polyp tissue and bone marrow) (Figure 6B).

### Transcription Factor Enrichment Analysis of Upregulated Genes in PO-MSCs and in BM-MSCs

**Figure 7:**
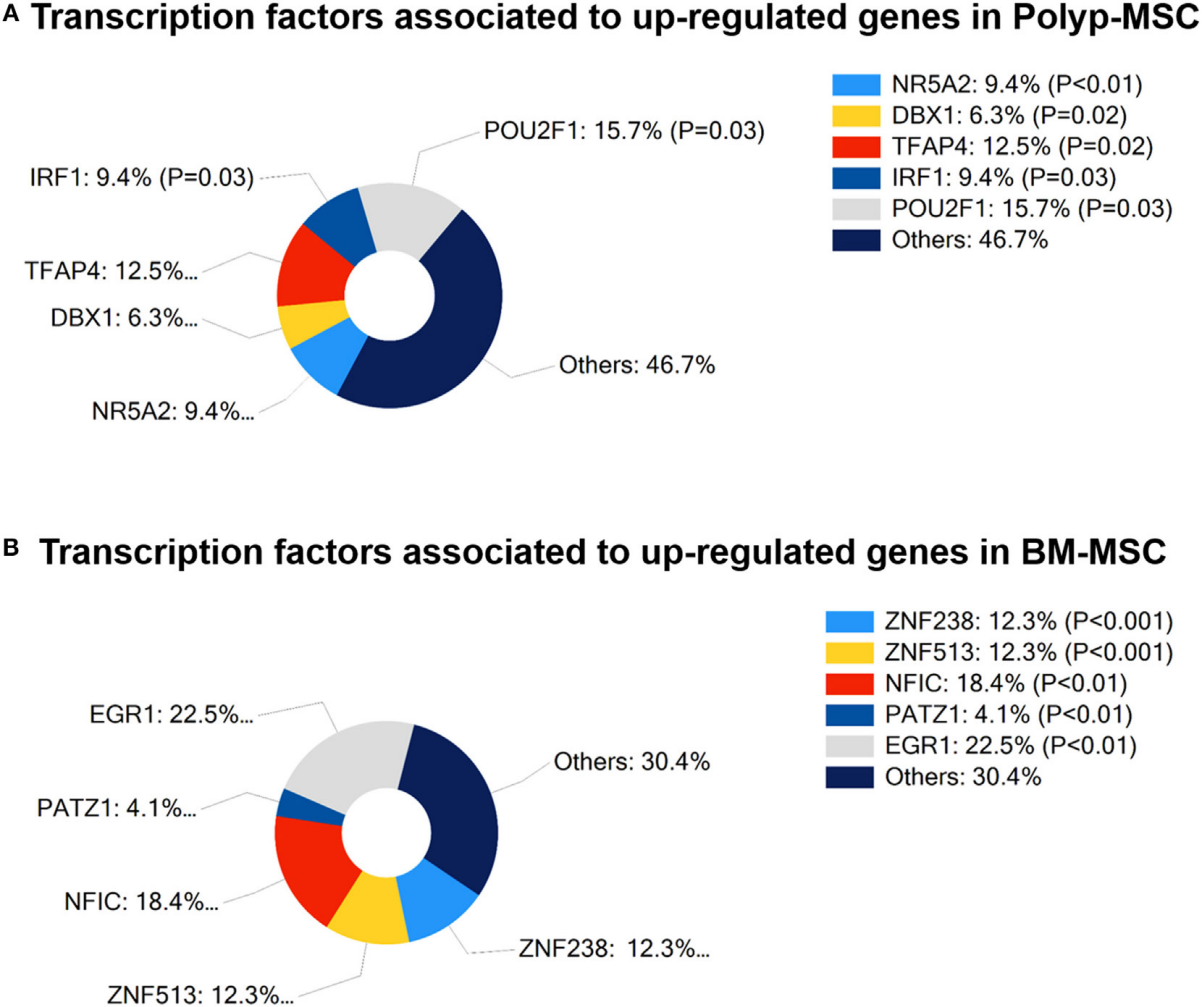
**Transcription factors associated with differentially modulated genes in Polyp-MSCs (PO-MSCs) and in bone marrow-derived MSCs (BM-MSCs)**. **(A)** Percentage of participation of transcriptional regulators in upregulated genes in PO-MSCs. **(B)** Contribution (%) of main transcriptional regulators associated with upregulated genes in BM-MSCs. It is possible to observe that the transcription factors most often associated with modulated genes in PO-MSC were POU2F1 (15.7%, *p* = 0.03) and TFAP4 (12.5%, *p* = 0.02), while EGR-1 (22.5%, *p* < 0.01) and NFIC (18.4, *p* < 0.01) were predicted as main transcriptional regulators in BM-MSC.

In search for functional regulatory association regarding upregulated genes in PO-MSC or in BM-MSC, we empirically pointed out the most often associated transcriptional factors that were enriched with these genes. PO-MSCs showed the representative participation of POU2F1 (15.7%, *p* = 0.03) and TFAP4 (12.5%, *p* = 0.02) as main transcriptional regulators at upregulated genes, which are related to cancer stem cells, cell cycle, and histone regulation (Figure 7A). On the other hand, the transcription factors most often associated with upregulated genes in BM-MSCs were EGR-1 (22.5%, *p* < 0.01) and NFIC (18.4%, *p* < 0.01), which are linked to cell proliferation, differentiation, apoptosis, and cell growth (Figure 7B). Thus, based on transcription-binding sites regulation pattern, these cells can represent different MSC subtypes, which modulate their transcriptional profile according to the distinct interaction with specific microenvironments in which these cells are found (polyp tissue and bone marrow).

## Discussion

Mesenchymal stromal cells can be isolated from almost all tissues and effectively cultured *in vitro*. Although their actual properties and biological functions have not been completely elucidated, these cells have been shown to represent an important approach in several clinical applications and in the regulation of immunocompetent cell responses. Human nasal-derived MSCs have been isolated mainly from inferior turbinate tissue and from olfactory tissue by several groups, and it has been suggested to have a valuable role in tissue engineering and regenerative medicine (15–20).

In this study, we have isolated MSCs from abnormal nasal polyp tissue (PO-MSC) and compared their gene expression profile and immune phenotype with BM-MSCs. Our results showed that several MSCs immune-associated membrane markers CD117, HLA-DR, PD-L1, and PD-L2 were not expressed in PO-MSCs but were expressively present in BM-MSCs. These markers are mainly associated with the immuneregulatory capacity of MSCs. For instance, PD-L1 molecule is a component of the T-lymphocyte costimulatory pathway and plays a crucial role in controlling T-cell proliferation and immunosuppression, and hence, it prevents tissue damage and autoimmunity (21, 22). Furthermore, PD-1 ligand 2 (PD-L2) may also inhibit T-cell receptor-mediated proliferation and cytokine production by CD4^+^-activated T-cells (23). PD-L1 and PD-L2 have been reported to be highly expressed by human placenta MSCs, which have a strong effect on adhesion, migration, and immunosuppression mechanisms of T-cells (24). Considering this perspective, we performed here a classical immunosuppressive coculture assay with both MSC subtypes (Polyp and BM) in contact with CD4^+^ and CD8^+^ lymphocytes. We observed that both MSC populations suppressed lymphocytes proliferation; however, PO-MSCs were less effective at higher dilutions. In addition, this PO-MSC also promotes reduced Treg expansion when compared with BM-MSC at same proportion. These results suggest that, unlikely BM-MSCs, PO-MSCs may have limited capacity to modulate T-cell-mediated immune response by a mechanism apparently mediated by low expression of immunoregulatory surface markers (i.e., PD-L1 and PD-L2). A previous study of our group showed this mechanism when coculture of MSCs and lymphocytes were seeded with anti-PD-L1 inhibitor, and no Treg expansion or lymphocyte repression was observed (25). Thus, all this finding raises questions regarding the presence of strong anti-inflammatory properties in PO-MSCs, as classically observed in BM-MSCs (11, 13). Moreover, we identified that PO-MSCs, in comparison with BM-MSCs, expressed very lower levels of HLA-DR surface marker. Although BM-MSCs already is known to present low levels of HLA-DR (26), this finding can suggest an additional immunogenic advantage for PO-MSCs, since HLA-DR is widely involved with tissue engraftment and rejection (27) and its low expression suggests an therapeutic advantage for MSC derived from nasal polyp tissue. In fact, we assume that this fundamental tolerating ability was not tested in this study, and this point needs to be investigated in further works involving tumorigenesis assays.

To analyze the molecular properties of PO-MSCs, we carried out a specific gene expression assay comprising the most relevant stem cell gene profile. We found a set of 15 genes that were statistically overexpressed in PO-MSCs, when compared to BM-MSCs. Interestingly, we observed that three genes (i.e., *PROM1, HNF1A*, and *BMP7*) had a higher fold-change index (>50-fold regulation) in PO-MSCs than in BM-MSCs. The *PROM1*, also known as CD133, is a gene that encodes a transmembrane glycoprotein, which is expressed in human hematopoietic stem cells and mouse neuroepithelial cells (28, 29). This molecule is considered a key biomarker for isolation and characterization of progenitor hematopoietic stem cells (30) and plays a crucial role in maintaining stem cell properties by suppressing cell differentiation and regulating crucial cellular events such as regeneration, differentiation, and metabolism. The *PROM1* and Nestin (*NES*) have been reported to be strongly expressed in ectomesenchymal stem cells derived from rat nasal respiratory mucosa (31). These genes are also highly expressed in cells from the nervous system, as observed by Weigmann et al. (29), suggesting that PO-MSCs may have a possible potential to differentiate into neuronal-like cells as nasal MSCs. Otherwise, this phenotype can also represent basal cells from the olfactory epithelium, which act as supportive stem cells for the neuroepithelial lineages and could be located inside of PO-MSC population, expressing higher levels of *PROM1*; however, this association needs also to be demonstrated in further investigations.

The *HNF1*, which was also upregulated in PO-MSCs, is a transcriptional activator that regulates the tissue-specific expression of multiple genes, especially in pancreatic islet and liver cells. *HNF1* is also included in the human embryonic stem cell pluripotency pathway, where it regulates the transcription of other genes involved in cell growth, cell adhesion, epithelial formation, immune system, and inflammation, including *TNF* (32). Additionally, *HNF1* may indirectly regulate the expression of *BMP7*, which encodes a complex of adherent junction proteins. These molecules are crucial for the formation and maintenance of epithelial cell layers, and hence, they participate in cell growth regulation (33). In NP context, the HNF1/BMP7 axis is important for epithelium integrity and is associated with *TGF-*β expression during the remodeling process (34–38). Altogether, these genes are closely associated with NP remodeling process, suggesting a prospective role of PO-MSCs in the modulation of NP microenvironment.

In our global expression gene analysis, we also detected four genes (i.e., *GDF6, KDR, FGF10*, and *GDF5*) that were significantly downregulated (more than 20-fold regulation) in the PO-MSCs when compared to BM-MSCs. The growth differentiation factors *GDF5* and *GDF6* are part of the TGF superfamily, which regulates cell proliferation and differentiation, bone and cartilage formation, and skeletal development. BM-MSCs have a strong capacity to differentiate into osteoblasts (39), and it is not surprising to find a broad expression of osteoblastic factors upregulated in these cells. However, the transcription level deficiency of *GDF5* and *GDF6* in PO-MSCs could not be totally clarified in this study. *GDF-5* has been shown to play a crucial role not only in several musculoskeletal processes (40, 41) in several studies but also in cell proliferation, inflammation, and extracellular matrix formation (42). The role of this factor in the expression of metalloproteinases and their inhibitors and the influence in healing during tendon injuries were also demonstrated by Park et al. (43). It has been also suggested that *GDF6* is involved in tissue vascular regeneration and angiogenesis through regulation of the expression and the signaling of Smads molecules (44). However, it is noteworthy mentioning that all this evidence is based on the results obtained from studies performed *in vitro* or in animal models, which use mainly exogenously administrated or recombinant GDFs isoforms. Hence, the exact role of these growth differentiation factors in PO-MSCs still remains unclear.

Complementary to gene expression profile, our *in silico* prediction analysis showed that POU2F1 and TFAP4 motifs are predicted to be enriched in PO-MSCs. These transcription regulators are mainly related to cancer stem cells, cell cycle, and histone regulation, attributing an internal transcriptional regulatory potential to MSC from NP tissue (45). On the other hand, we found EGR-1 and NFIC as predicted transcription factors to be regulated in BM-MSCs. These molecules are linked to cell proliferation, differentiation, apoptosis, and growth, suggesting that bone marrow MSC has a prominent role as a cellular regulator, mainly through the secretion of wide range of growth factors (46, 47).

Considering this evidence, we have demonstrated that although PO-MSCs and BM-MSCs share some features (i.e., morphology, surfaces markers, and differentiation capacity), they have a different transcriptional regulation profile and possibly distinct physiological function. Since that polyp is an abnormal tissues that grow over chronic inflammatory condition (3), this observation is in line with a previous study showing that human MSC from ethmoid sinus mucosa has different proliferation capability and distinct expression of immunomodulatory cytokines when compared to MSCs isolated from the maxillary sinus, ethmoidal mucosa, and inferior turbinate (15); these findings support the idea that the inferior turbinate mucosa has different molecular, cellular, and mechanical properties, when compared with middle meatus mucosa (8). Similarly, as expected, our study also suggests that MSC from abnormal polyp nasal mucosa has distinct molecular pattern in comparison with MSCs from normal bone marrow tissues.

Recently, two studies reported the isolation of MSCs from nasal polyp tissue and their results completely support our findings (14, 15). In addition, in our study here presented we went one step further and provided support to the concept that MSCs may have inherent properties and a gene/protein expression pattern according to their anatomical location and their interaction with specific microenvironments. It is remarkable that the most significant differentially expressed genes in PO-MSCs were partially linked to metabolic and ECM/tissue remodeling/regeneration processes, which are signaling pathways clearly affected in nasal polyp disease (48). Nevertheless, considering PO-MSCs as a possible therapeutic alternative for tissue regeneration, we need to know the implications of this gene expression profile *in vivo*, as well as clarify the intrinsic association of these cells with their pro-inflammatory environment.

In conclusion, in this study, we compared two MSC populations and demonstrated the lack of immunoregulatory markers and a different transcriptional profile in PO-MSC in comparison to BM-MSCs, suggesting that this distinct molecular pattern may be indirectly implicated in the development of nasal polyp tissue.

## Author Contributions

Conceptualization: PW and RP. Investigation and resources: PW, RP, JA, CP-N, WB, and DA. Supervision: RP, CP-N, WB, NC, and LG. Writing: RP, DA, and CP-N.

## Conflict of Interest Statement

The authors declare that the research was conducted in the absence of any commercial or financial relationships that could be construed as a potential conflict of interest.
