# Supplementary material for: Nasal Polyp-Derived Mesenchymal Stromal Cells Exhibit Lack of Immune-Associated Molecules and High Levels of Stem/Progenitor Cells Markers
Source: Front Immunol. 2017 Jan 30;8:39. doi: 10.3389/fimmu.2017.00039 (PMC5276864; doi:10.3389/fimmu.2017.00039)
Supplement: Supplementary file 1 [file Presentation_1.PDF]

Supplementary Information

TITLE: Comparison of *in vitro* features and gene expression profile of polyp-derived mesenchymal stem cells and bone marrow-derived mesenchymal stem cells.

Pedro Oliveira Wey, Rogério Pezato, Juan Sebastian Henao Agudelo, Claudina Angela Perez-Novo, Wim Vanden Berghe, Niels Olsen Saraiva Câmara, Danilo Candido de Almeida, Luís Carlos Gregorio.

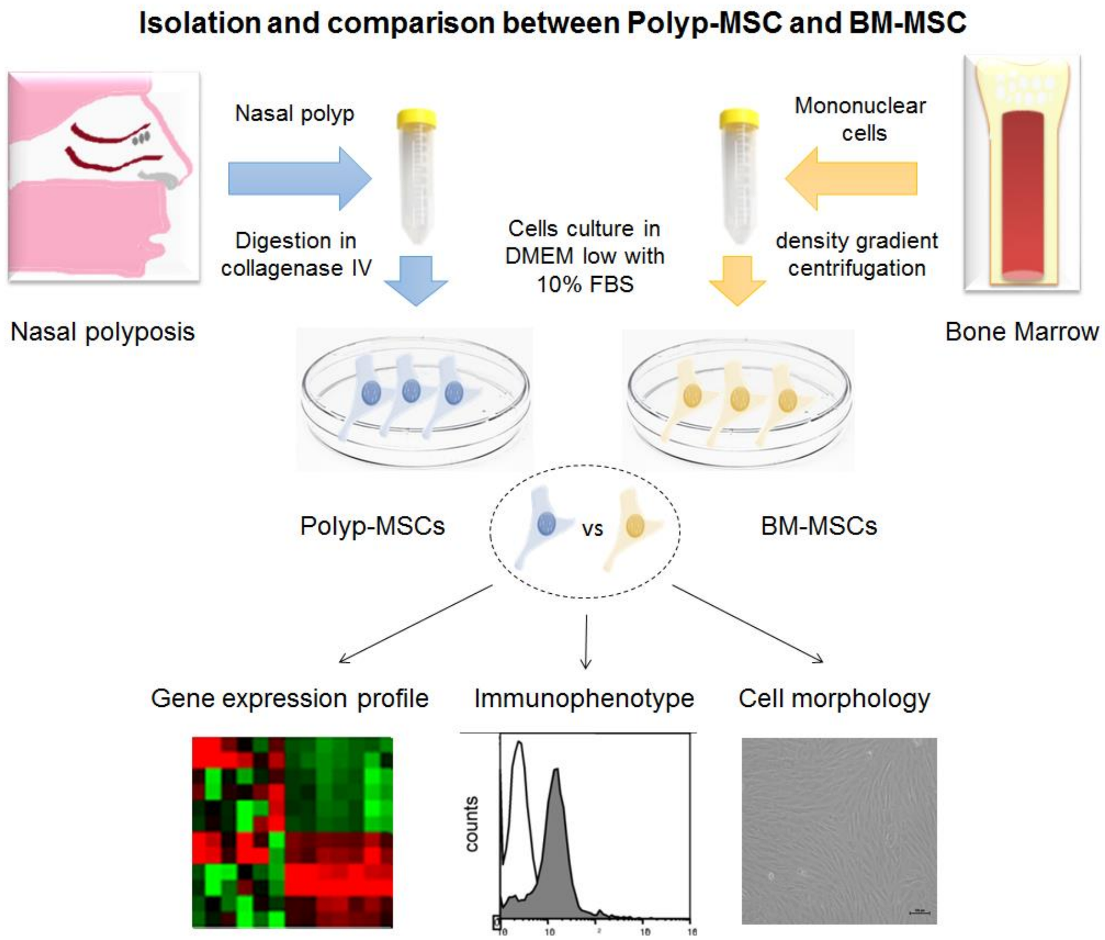

Figure S1. An overview of experimental design for isolation and comparison of Polyp-MSCs and BM-MSCs. The Polyp-MSCs were obtained from polyps derived from patients with nasal polyposis and submitted to mechanic and enzymatic dissociation. The BM-MSCs were isolated by density-gradient from bone marrow of health individuals. Both MSCs subsets were cultivated in culture flasks containing DMEM-Low with 10 % FBS. Then, the cells were evaluated concerning their morphological, transcriptional and protein surface aspects.

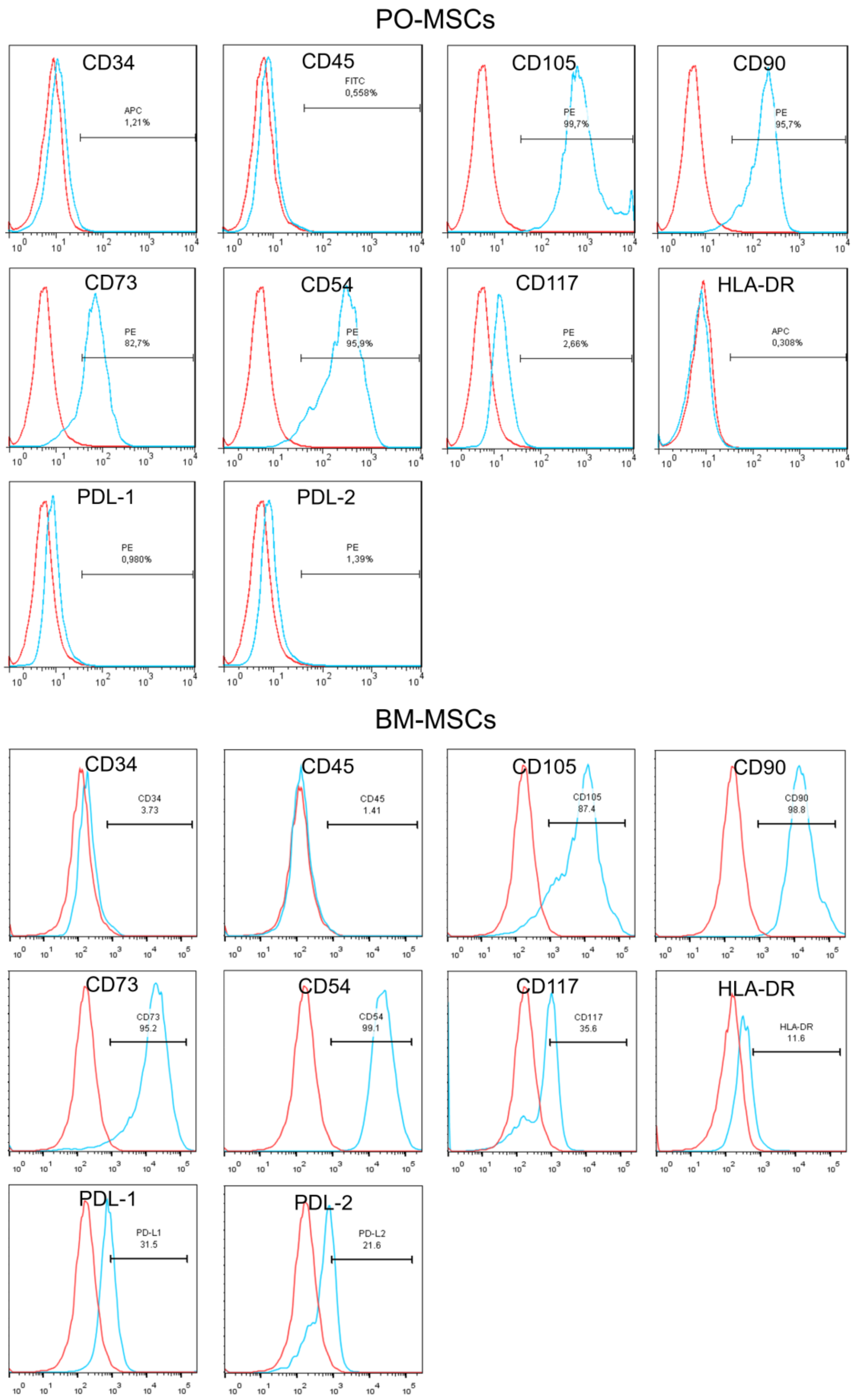

Figure S2. Immunophenotype profile of PO-MSCs and BM-MSCs. Polyp-MSCs and BM-MSCs were characterized conforming its immunophenotyped profile. It was observed that both MSCs subsets share the same pattern, but PO-MSCs have low expression of HLA-DR, PDL-1 and PDL-2 markers.

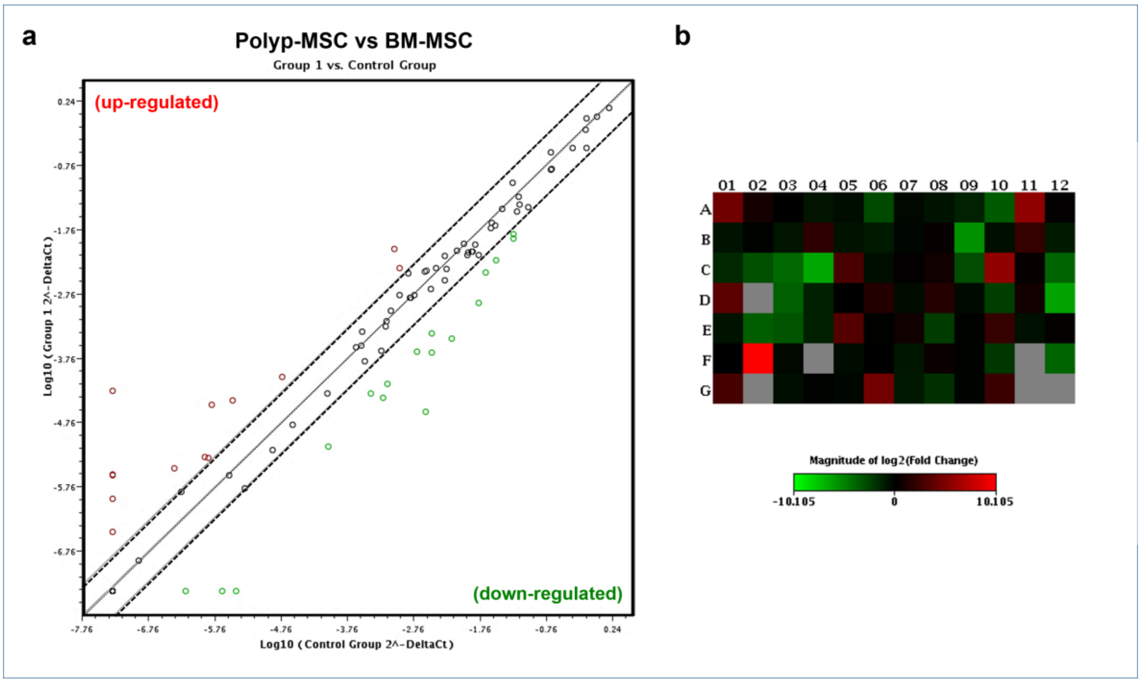

Figure S3. Scatter-plot analysis and plate heat map. (**a**) The scatter-plot analysis showing the up- and down regulated genes with 2-fold change in the comparison Polyp-MSCs versus BM-MSCs. (**b**) Heat map of PCR 96 well plate illustrating the most significant regions (green and red) in the plate which the genes were modulated in Polyp-MSCs when compared with BM-MSCs.

Table S1. Individual phenotype profile of MSCs samples.

markers	BM-MSC 1	BM-MSC 2	BM-MSC 3	BM-MSC 4	BM-MSC 5	BM-MSC 6	Polyp-MSC 1	Polyp-MSC 2	Polyp-MSC 3
CD34	6.92	5.44	4.43	4.612	4.896	3.7212	1.21	0.98	0.35
CD45	1.98	1.25	0.87	2.178	1.125	0.7266	0.58	0.74	0.41
CD105	74.40	82.15	76.53	81.84	73.935	64.281	99.70	99.40	97.80
CD90	96.50	98.70	95.85	96.15	88.83	80.514	95.70	96.70	94.45
CD73	90.80	92.30	89.80	99.88	83.07	75.432	82.70	85.22	86.70
CD54	92.40	91.40	90.15	99.64	82.26	75.726	95.90	92.90	92.65
CD117	31.40	28.70	28.30	24.54	25.83	23.772	2.66	3.68	1.42
HLA-DR	20.10	13.40	15.00	9.3	8.80	11.20	0.30	0.57	0.19
PDL-1	33.20	32.60	31.15	36.52	29.34	3.7212	0.98	1.23	0.36
PDL-2	26.60	28.20	25.65	29.26	25.38	0.7266	1.39	1.76	0.83
